# Auditory brainstem mechanisms likely compensate for self-imposed peripheral inhibition

**DOI:** 10.1038/s41598-023-39850-8

**Published:** 2023-08-04

**Authors:** Sriram Boothalingam, Abigayle Peterson, Lindsey Powell, Vijayalakshmi Easwar

**Affiliations:** 1 Waisman Center and Department of Communication Sciences and Disorders, University of Wisconsin-Madison, Madison, WI 53705 USA; 2 Macquarie University, Sydney, NSW 2109 Australia; 3 National Acoustic Laboratories, Sydney, NSW 2109 Australia

**Keywords:** Auditory system, Sensory processing

## Abstract

Feedback networks in the brain regulate downstream auditory function as peripheral as the cochlea. However, the upstream neural consequences of this peripheral regulation are less understood. For instance, the medial olivocochlear reflex (MOCR) in the brainstem causes putative attenuation of responses generated in the cochlea and cortex, but those generated in the brainstem are perplexingly unaffected. Based on known neural circuitry, we hypothesized that the inhibition of peripheral input is compensated for by positive feedback in the brainstem over time. We predicted that the inhibition could be captured at the brainstem with shorter (1.5 s) than previously employed long duration (240 s) stimuli where this inhibition is likely compensated for. Results from 16 normal-hearing human listeners support our hypothesis in that when the MOCR is activated, there is a robust reduction of responses generated at the periphery, brainstem, and cortex for short-duration stimuli. Such inhibition at the brainstem, however, diminishes for long-duration stimuli suggesting some compensatory mechanisms at play. Our findings provide a novel non-invasive window into potential gain compensation mechanisms in the brainstem that may have implications for auditory disorders such as tinnitus. Our methodology will be useful in the evaluation of efferent function in individuals with hearing loss.

## Introduction

Efferent neural networks fine-tune and regulate afferent sensory inputs. One such network at the level of the auditory brainstem, the medial olivocochlear reflex (MOCR), modulates activity at the most peripheral level, the cochlear outer hair cells (OHCs). The OHCs actively amplify basilar membrane motion for low-level sounds. When activated, the MOCR inhibits this amplification process, thus turning down the cochlear gain. This reduction in cochlear activity is thought to be useful for signal detection in noise^1,2^ and protection against loud sounds^3–5^. While the peripheral consequences of this inhibition are well-understood from studies using measures such as otoacoustic emissions (OAEs) and auditory nerve compound action potentials (CAPs), the upstream neural influence remains unknown, especially in humans^6–8^. As such, the goal of this study was to determine the central consequences of peripheral MOCR inhibition. Our motivation stems from the need to uncover the ecological relevance of MOCR inhibition. This requires an improved understanding of its effect along the entire auditory pathway at different timescales.

It is well-established that stimulus-driven peripheral responses such as CAPs and OAEs undergo robust attenuation in laboratory animals when the MOCR is activated^2,9–13^. However, current evidence perplexingly exhibits a disparity of MOCR influence in the central systems based on the presumed location of response generation along the auditory neuraxis in humans. For instance, endogenous components of cortical responses (e.g., auditory steady-state responses [ASSR] elicited at 40 Hz and thalamocortical loop resonance in the gamma band), undergo considerable attenuation in the presence of putative MOCR activation^14–20^. However, sensory-driven neural responses that originate in the brainstem (e.g., ASSR elicited at 80 Hz and auditory brainstem response [ABR] wave V), appear unaffected under the same testing conditions^14,15^. This brainstem immunity to MOCR effects, typically elicited by contralateral noise, remains unexplained. Here, we seek to clarify our hypothesis that brainstem-dominant neural responses show immunity because local feedback networks in the brainstem compensate for peripheral inhibition.

**Figure 1:**
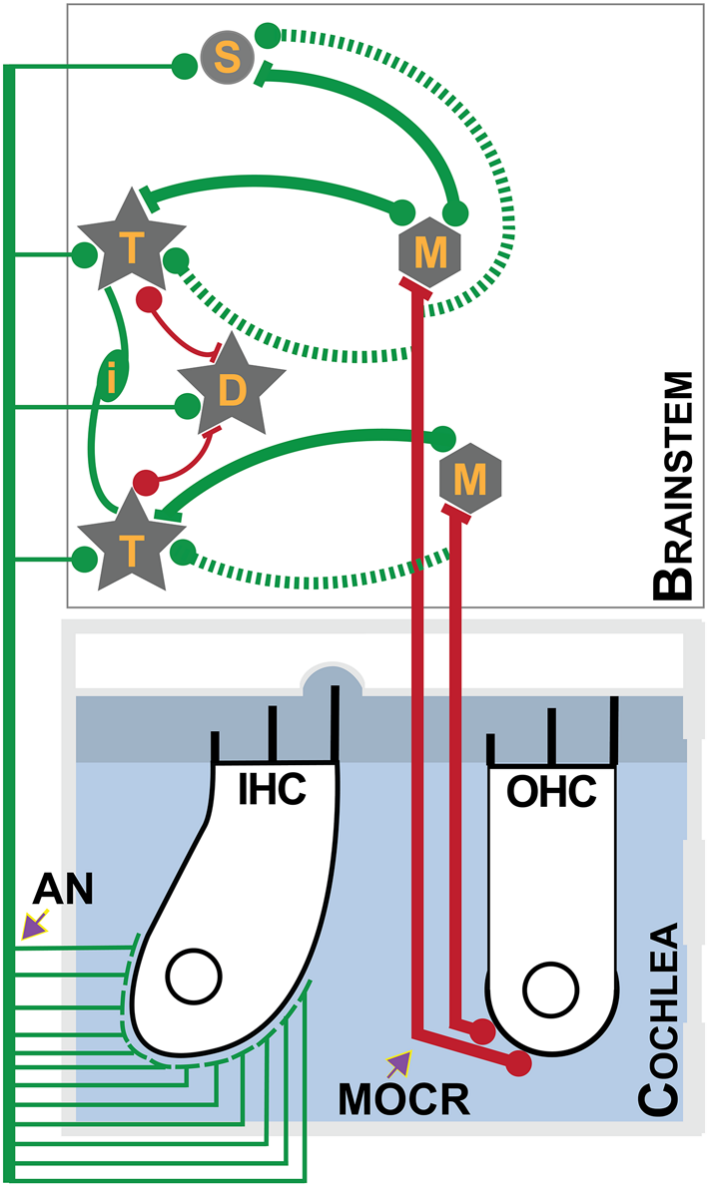
Schematic of a compensatory circuit in the brainstem. Known neural connections between the cochlea and the brainstem and between neuron types within the brainstem are schematically shown. Green lines indicate excitatory and red lines indicate inhibitory inputs. Axon terminals on their synaptic targets are indicated by filled circles implying the direction of information flow. Inputs from the inner hair cells (IHC) are distributed in the cochlear nucleus to T-stellate (T) cells, D-stellate (D) cells, and small (S) cells by the auditory nerve (AN). D-stellate cells provide inhibitory inputs to the T-stellate cells, and pairs (possibly more) of the T-stellate cells are interconnected via an unknown excitatory interneuron (i). Both T-stellate and small cells project to the MOC (M) neurons at the level of the superior olivary complex. Both T-stellate and small cells in turn receive excitatory collaterals (dashed lines) from the MOC *en route* to the outer hair cells (OHC)—the putative efference copy circuit.

**Figure 2:**
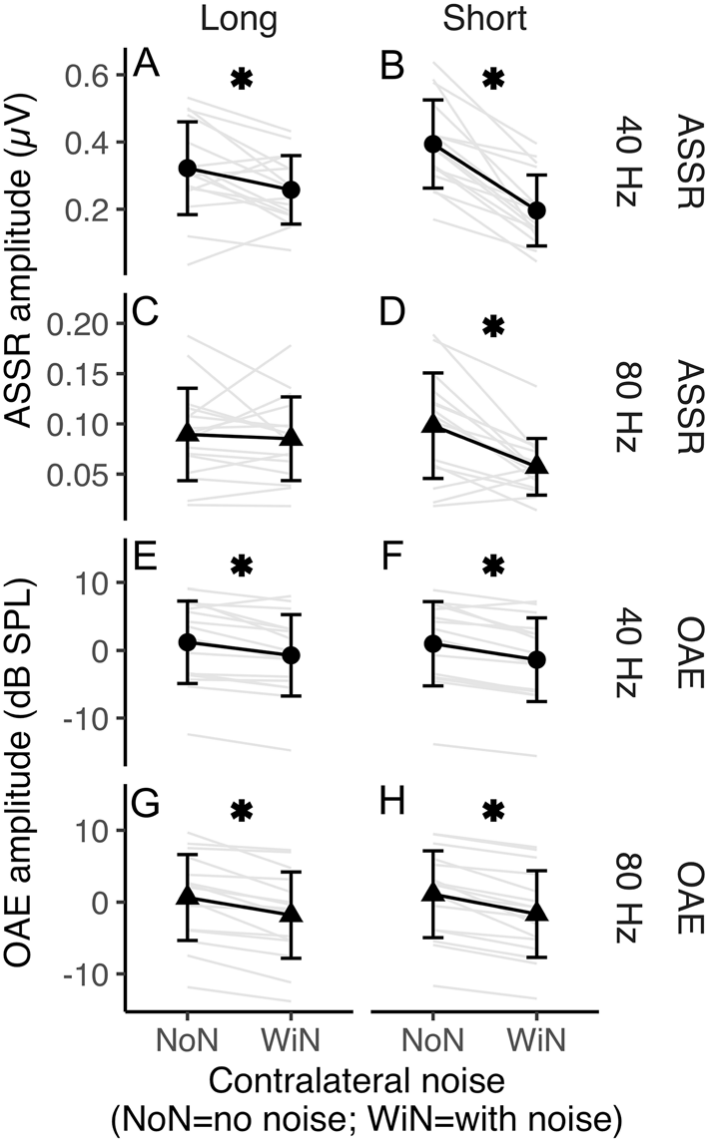
Response amplitude change with contralateral noise. ASSR in the top four panels and OAEs in the bottom four panels. Columns separate long- and short-duration conditions and rows separate 40 and 80 Hz click rates. Black circles (40 Hz click rate) and black triangles (80 Hz click rate) indicate group means and grey lines represent individual participants. Error bars represent ± one standard deviation. Asterisks denote a significant difference in amplitude between with- (WiN) and no-noise (NoN) conditions.

The rationale for our hypothesis is rooted in (1) previously identified circuits that are capable of such compensation^21–24^, as illustrated in Fig. 1, and (2) gain compensation that occurs for more extreme peripheral input loss due to pathologies such as cochlear ablation, deafferentation, and conductive deficits^25–27^. The brainstem circuit capable of compensation for peripheral inhibition involves a positive feedback loop between T-stellate and small cells in the cochlear nucleus and the MOC neurons. As illustrated in Fig. 1, not only do both T-stellate and small cells provide primary excitatory inputs to the MOC, but they also receive excitatory collaterals back from the MOC *en route* to the cochlea. This arrangement means the neurons that excite the MOC are excited in return, possibly to the same degree as the peripheral inhibition, similar to the concept of efference copy in the motor cortex^28,29^. This local MOC-mediated compensation may traverse rostrally and (1) sequentially through multiple intermediate nuclei, as well as (2) through their direct inputs, to the inferior colliculus^30^. Either way, the compensation at the cochlear nucleus has the potential to influence evoked potentials such as the ASSR and ABR generated in the brainstem/midbrain. We predict that if these local feedback networks do compensate for the MOCR-mediated peripheral inhibition it would be expected to occur over a period of time, i.e., with a latency relative to stimulus presentation. Based on the time it takes for the MOCR inhibition on OHCs to stabilize (~ 400 ms^31,32^), we speculate that the time taken for complete compensation will be between 0.5 and a few seconds. To test this prediction, we concurrently measured peripheral (cochlear) and neural (brainstem or cortical) responses to short (1.5 s) and long (240 s or 4 min) click-trains with and without contralateral acoustic stimulation to activate the MOCR. Our results support our hypothesis in that, responses at the periphery (OAEs at 40 and 80 Hz), brainstem (80 Hz ASSRs), and cortex (40 Hz ASSRs) demonstrate robust inhibition in the short-duration condition, however, for the long-duration condition, only the inhibition of brainstem responses diminishes. That is, the inhibition appears to be compensated for at the brainstem between 1.5 s and 240 s. This approach likely provides a window into brainstem feedback circuits that are involved in enhancing the peaks of complex signals (e.g., speech) and possibly maintaining homeostasis in response to a reduction in auditory input from the periphery^21–24,28^. Our non-invasive approach, in addition to the cochlear microphonics^33–35^, may offer a solution to evaluating auditory efferent function in patients with sensorineural hearing loss and offers a new perspective on the ecological relevance of the MOCR.

## Results

ASSR and OAE amplitudes are plotted as a function of click rate (40 vs. 80 Hz), contralateral noise (with vs. without), and duration (long vs. short) in Fig. 2. The reduction in OAE magnitude appears relatively unchanged between short and long conditions unlike for ASSR magnitude where there are some differences between durations. A 3-way repeated measures analysis of variance (ANOVA) for ASSRs revealed a significant three-way interaction. That is, the effect of noise varied as a function of duration and rate (*F*[1, 15]) = 27.98*, p* < 0.001). Post-hoc *t*-tests were corrected for multiple comparisons using the False Discovery Rate approach (FDR^36^). We report FDR-corrected *p*-values and hence *p* < 0.05 are to be interpreted as significant. These post-hoc tests demonstrate a significant effect of noise on 40 Hz ASSR in both short (*p* < 0.001; Fig. 2B) and long (*p* = 0.03; Fig. 2A) durations, however, the effect of noise on 80 Hz ASSR was only significant for the short (*p* = 0.002; Fig. 2D) but not long (*p* = 0.651; Fig. 2C) duration.

A 3-way repeated measures ANOVA on OAE amplitude showed no significant 3-way interaction but significant two-way interactions between duration and click rate (*F*[1, 15] = 8.8*, p* = 0.009) and between duration and noise (*F*[1, 15] = 5.32*, p* = 0.036). Further, OAE amplitude also varied as a main effect of noise (*F*[1, 15] = 62.64*, p* < 0.001) suggesting peripheral inhibition through MOCR activation, as expected. Post-hoc *t*-tests suggest the effect of noise on OAE amplitude was significant for both short (*p* < 0.001) and long (*p* < 0.001) durations when averaged over both rates.

**Figure 3:**
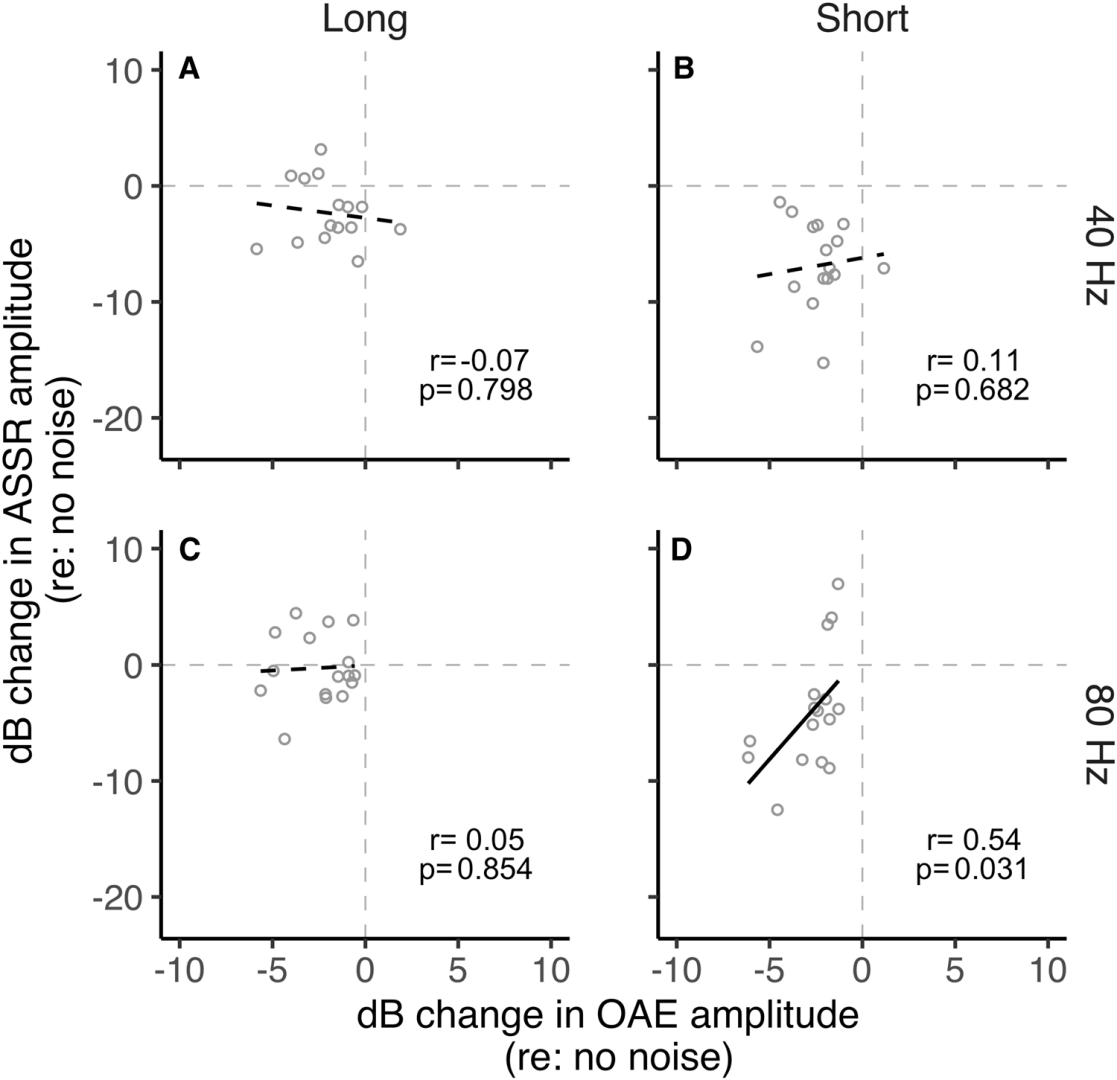
ASSRs vs. OAE amplitude change. (**A**) 40 Hz click-rate, long stimulus duration (**B**) 40 Hz click-rate, short stimulus duration (**C**) 80 Hz click-rate, long stimulus duration (**D**) 80 Hz click-rate, short stimulus duration. Open circles represent individual participants. A black solid fit line represents a significant relationship between variables. A black dashed fit line represents a nonsignificant relationship between variables.

To investigate the influence of MOCR inhibition in the cochlea (OAEs) on the inhibition at the brainstem (80 Hz ASSR) and cortical (40 Hz ASSR) levels, we performed correlations. These relationships are plotted in Fig. 3. ASSR and OAE inhibition were not correlated for the 40 Hz short condition (*p* = 0.682), the 40 Hz long condition (*p* = 0.798), and the 80 Hz long condition (*p* = 0.84). However, the change in OAEs and ASSRs was positively correlated in the 80 Hz short-duration condition (*p* = 0.031; Fig. 3D) with a moderate-to-large effect (*r* = 0.54)^37^. Further, the trend in the data (Fig. 3D) is quite apparent, unlike the other non-significant correlations. This result likely suggests that changes observed in ASSRs generated predominantly at the cortex (40 Hz) are likely not influenced by MOCR-induced OAE changes at the periphery. However, for ASSRs generated predominantly at the brainstem (80 Hz), the changes in the cochlea likely influence neural activity when viewed in shorter time intervals, but this washes out when observed over a longer time window.

**Figure 4:**
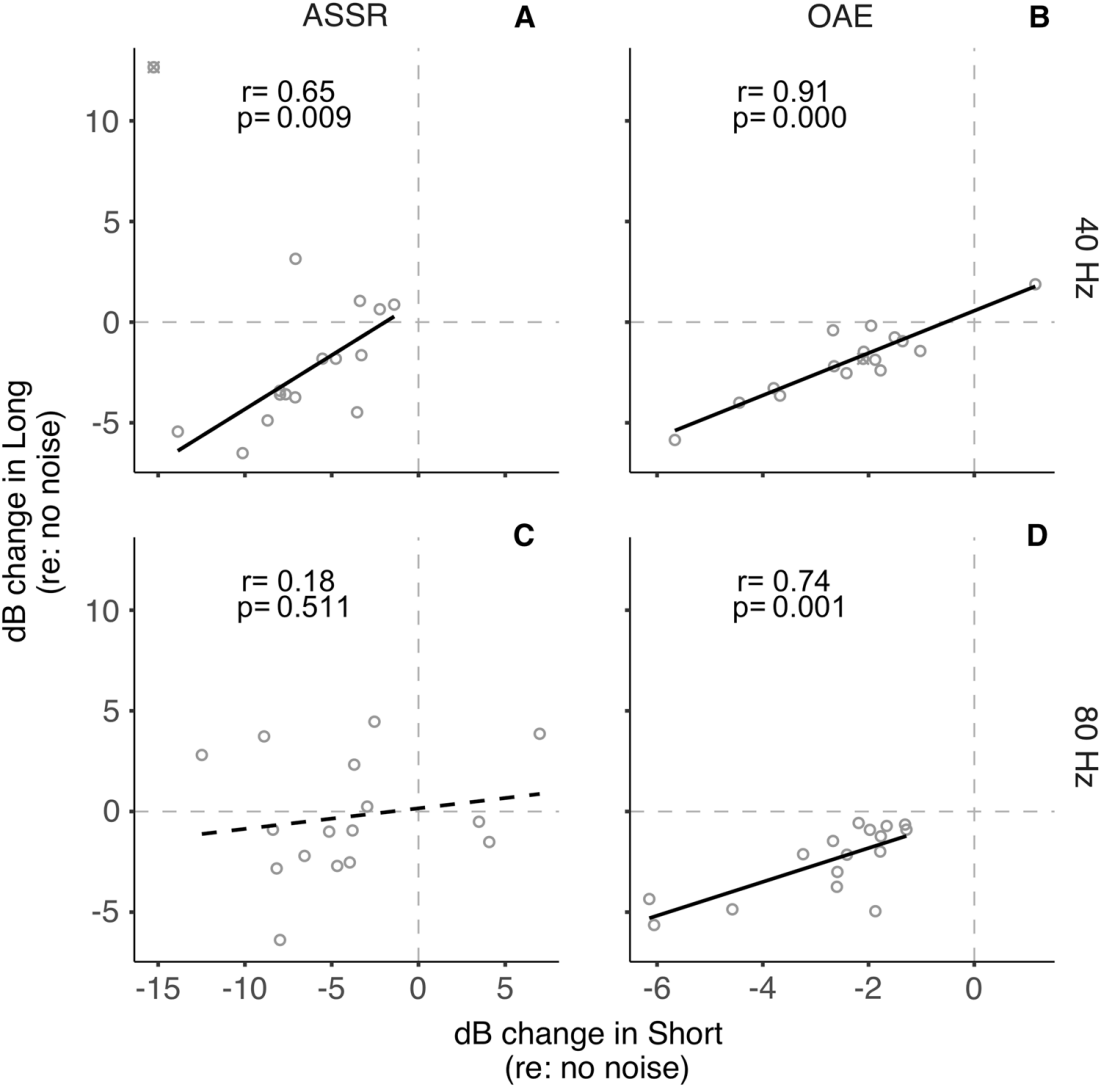
Long vs. short duration. Amplitude changes in the long stimulus durations as a function of amplitude change in the short stimulus durations are plotted for (**A**) 40 Hz click-rate, ASSRs (**B**) 40 Hz click-rate, OAEs (**C**) 80 Hz click-rate, ASSRs (**D**) 80 Hz click-rate, OAEs. Open circles represent individual participants. A black solid regression line indicates a significant relationship between the two variables. A black dashed regression line indicates a non-significant relationship between the two variables. An outlier in panel-A, not included in the correlation, is indicated with an additional ‘X’ symbol.

The relationship between the short- and long-duration conditions for ASSRs and OAEs in the magnitude of inhibition is shown in Fig. 4. There was a significant positive correlation between OAE inhibition in short and long durations observed for both 40 Hz (*p* < 0.001; Fig. 4B) and 80 Hz (*p* = 0.001; Fig. 4D) rates. This indicates that individuals exhibited similar OAE inhibition magnitudes in long and short durations, likely due to a single mechanism, the MOCR, influencing activity in the cochlea. For the 40 Hz ASSR, when the one outlier (cross symbol in Fig. 4A) is excluded from the analysis, we observe a significant positive correlation, consistent with visual inspection of the relationship (*p* = 0.009; Fig. 4A). This correlation is not significant if the outlier is included (*p* = 0.558). However, such a relationship was not observed for the ASSR inhibition at 80 Hz (*p* = 0.511; Fig. 4C), suggesting differential effects of the eliciting contralateral noise depending on the duration of the stimulus.

**Figure 5:**
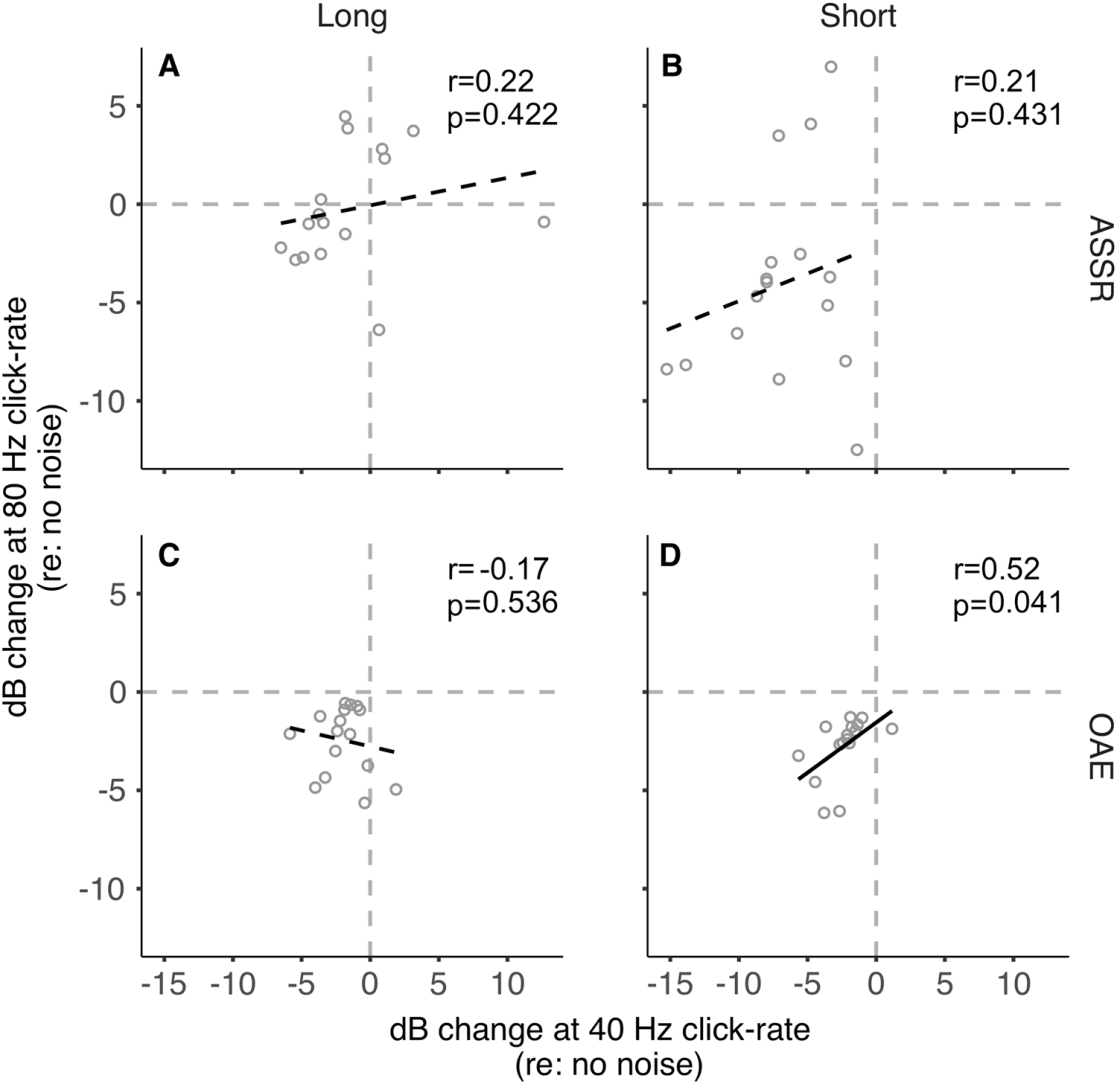
40 Hz vs. 80 Hz. Amplitude changes at 80 Hz click-rate as a function of amplitude change at 40 Hz click-rate for (**A**) ASSRs, long stimulus duration (**B**) ASSRs, short stimulus duration (**C**) OAEs, long stimulus duration (**D**) ASSRs, short stimulus duration. Open circles represent individual participants. A black solid regression line indicates a significant relationship between the two variables. A black dashed regression line indicates a non-significant relationship between the two variables.

We also compared inhibition between the two click rates within short and long durations separately for ASSRs and OAEs. This was to test if responses generated at various levels of the auditory pathway are related in the manner they are measured in this study. The relationships are plotted in Fig. 5. For ASSRs, there was no correlation between the inhibition at both click rates for short (*p* = 0.431; Fig. 5B) and long (*p* = 0.422; Fig. 5A) durations, as expected. This likely indicates that the amplitude changes observed in cortical-dominated (40 Hz) ASSRs are likely independent of brainstem-dominated (80 Hz) ASSR changes in both duration conditions. For OAEs, while there was a significant correlation between 40 and 80 Hz in the short condition (*p* = 0.041; Fig. 5D), the correlation in the long condition was not significant (*p* = 0.536; Fig. 5C), consistent with the interaction between rate and duration in the ANOVA for OAEs. It is unclear what this single inconsistent finding in OAEs means. Despite no objective evidence for a systematic MEMR influence on any MOCR estimates (see MEMR sub-section in Methods), small MEMR effects potentially influencing this condition cannot be categorically ruled out. However, when considered collectively, these results align with our hypothesis that brainstem mechanisms likely compensate for self-imposed peripheral inhibition.

## Discussion

Our primary finding is that neural responses predominantly generated at the brainstem do indeed undergo inhibition when analyzed in short intervals only—a time-sensitive effect that was not observed for cochlear- and cortical-evoked responses. Our results are consistent with, and expand the findings of, previous studies reporting similar findings that use long duration stimuli^14–20^.

### What causes the discrepancy in inhibition between the brainstem and the cortex?

Given the lack of methodological differences, and similarity in peripheral MOCR inhibition between duration conditions, the differential stimulus duration effect of noise on the 80 Hz, but not 40 Hz ASSR, can be conjectured to arise from the physiological differences in brainstem vs. cortical response to contralateral noise. Previous studies that demonstrate similar generator-specific effects of contralateral noise speculate that contralateral noise only affects the functioning of structures rostral to the 80 Hz ASSR generators^14,16^. As such, any peripheral inhibition caused by the MOCR is insignificant for retrocochlear neural responses^14^. Does this mean the MOCR inhibition is in vain for brainstem neural systems? The short answer to this question is *no* because attenuation of auditory nerve compound action potentials is an established marker of MOCR activity in the afferent auditory pathway in animal models^2,9–12^. Further, the primary finding of this study, a robust reduction of 80 Hz ASSR for short-duration contralateral noise, provides ample evidence that MOCR inhibitory effects persist well beyond the cochlea and into the brainstem. The question then is how is this inhibition not observed when averaged over longer time intervals (mins) in the brainstem, and how does it persist in the cortex? Below we discuss a hypothesis rooted in the physiology of known feedback circuits in the brainstem along with other alternative possibilities.

#### Brainstem circuits may compensate for MOCR-mediated peripheral inhibition

Our results may, for the first time, provide reasoning for the perplexing “non-significant” effect of the MOCR on brainstem-generated evoked potentials. Consistent with our hypothesis, we posit that the MOCR-mediated peripheral inhibition probably *does* become insignificant at the brainstem because mechanisms in the brainstem compensate for the loss in input at the periphery over a few seconds. Based on positive feedback loops between the cochlear nucleus and the superior olivary complex identified in previous animal studies, the likely candidates for such compensation would be the T-stellate^21–23^ and the small cells^24^ as illustrated in Fig. 1. Both T-stellate and small cells of the cochlear nucleus receive direct inputs from the auditory nerve^38,39^, project to MOC neurons^40–44^, and receive collaterals from MOC neurons^21–24,45–47^. As schematically shown in Fig. 1, these projections and their inputs create positive feedback loops for both cell types (i.e., T-stellate-MOC-T-stellate and small cell-MOC-small cell) ideal for compensation of MOCR-imposed peripheral inhibition. The enhancements in ASSR amplitude with contralateral noise, re: no-noise (5/16 participants), predominantly seen in the 80 Hz long condition in Fig. 2C, albeit speculative, potentially adds further credibility to our hypothesis, in that these compensatory mechanisms may sometimes overshoot in their amount of compensation. Additionally, the MOC neurons also project rostrally to the inferior colliculus, a major site of generation for the 80 Hz ASSR and wave-V of the ABR, where they both increase and decrease the firing rate of different neuronal types as a function of input level^30,48^. The MOC neurons, therefore, have the potential to compensate for their self-imposed peripheral inhibition either through (1) the aforementioned cochlear nucleus feedback circuits, (2) directly influencing the inferior colliculus neurons, (3) a combination of influencing both the cochlear nucleus and inferior colliculus neurons, and/or (4) a different, currently unknown, pathway where the MOC projects.

#### Adaptation may restore brainstem-generated ASSR

Several previous reports, both in animal models (physiology) and in humans (psychoacoustics) highlight that neurons, including the auditory nerve, adapt their firing rate to the dominant ongoing stimulus statistics leading to an improvement in their dynamic range of firing^49–53^. This dynamic range adaptation exhibits varying time scales and may be influenced by the location within the auditory pathway, potentially manifesting as longer durations at higher levels^49,53^. Dynamic range adaptation can only be observed in paradigms where there is sufficient history for the neurons to learn and adapt. Short-term stimuli that lack a meaningful amount of history due to intermittent silence periods, that wash out recent stimulus information, do not lend themselves well to adaptation^49–53^. The contralateral noise effects observed for the 80 Hz ASSR in our study appear similar to such long-term-only adaptation effects. Furthermore, recent studies have implicated a role for the MOCR in such dynamic range adaptation^54^ and suggest differential, stimulus-specific, inhibition at different stages of the auditory pathway^55^. Given the direct influence of the cortex and midbrain on the MOCR, similar adaptive processes may be speculated to be in action for the compensation for 80 Hz ASSR in the long condition but not in the short condition. That is, in the long condition, putative adaptive mechanisms might cause an increase in gain leading to the restoration of ASSR amplitude. Although a similar phenomenon has been reported for spectrotemporal contrast adaptation in the cortex^56^, it should be emphasized that this conjecture is highly speculative, especially considering the 40 Hz ASSR does not undergo similar adaptation, and further studies are required to test this hypothesis (see also Discussion sub-section“If the brainstem compensates, why is inhibition still present in the brainstem and the cortex?”).

#### Slow effects in the efferents

Another potential possibility that may contribute to our observed differences is the MOCR slow effect observed in guinea pigs^57–59^. Although it is not readily observed in humans^60^, the slow MOCR effects are observed only when basilar membrane motion is measured continuously over tens of seconds. The slow effects are thought to be due to the MOCR causing greater axial stiffness of the OHCs as opposed to hyperpolarization as seen in the fast effects^57,58^. If present, slow effects may only be observed in our long-duration condition. However, a strong argument for a lack of such an effect in our data is that the inhibition of OAEs does not change as a function of stimulus duration. If slow effects were at play, we would expect (1) a difference in OAE inhibition between short- and long-duration conditions, which is not the case in our data, and (2) given the dual action of the MOCR across longer time scales, a larger reduction in ASSR amplitude would be expected for the long duration, which is also not the case in our data.

Although a recent study reported slow effects in their MEMR data^61^, considering (1) our data is dominated by the MOCR effects (see MEMR sub-section under Methods), and (2) because no difference in OAE inhibition is observed between short- and long-duration conditions, it is unlikely that any slow MEMR or changes in middle ear status, including pressure build-up over the course of the long duration condition, may explain our results.

### What could be the relevance of a compensatory circuit in the brainstem?

The positive MOCR feedback loops for both cell types, the T-stellate and small cells, are thought to act as ‘efference copies’ of MOCR inhibition at the periphery and likely compensate for the reduced input^21–24,28,29^. This gain compensation could be critical for at least three reasons. First, a reduction of input at the periphery will decrease excitation of the MOC and the facial motor neurons (MEMR), which will, in turn, limit their functional ability to protect vulnerable cochlear hair cells from acoustic overexposure^3–5^. Second, a reduction of input at the periphery might negatively impact central gain, and possibly the tuning of the T-stellate cells^62^ resulting from altered input to the D-stellate cells. As illustrated in Fig. 1, by providing inhibitory input to the T-stellate cells, the D-stellates help maintain a balance in gain in the cochlear nucleus among their various other functions^62–64^. Reduced T-stellate cell inhibition, combined with the recently discovered excitatory loop gain within T-stellate cells^65^ could lead to tinnitus and hyperacusis when left unchecked^24,65^. Third, compensating for reduced peripheral input likely restores, and possibly enhances, the fidelity of the sound level, specifically the spectral peaks as encoded by T-stellate cells^38,66,67^ and small cells^24^. At the population level, both T-stellate cells and small cells encode spectral peaks and have been identified to be critical for speech perception^24,38,66,67^. The MOC neurons only provide cholinergic input to the T-stellate, not D-stellate cells, which is thought to lead to selective enhancement of spectral peaks and not valleys, improving the overall SNR in the system^21^. Further, T-stellate and small cells respond optimally at moderate to high stimulus levels, like the elicitor used in the present study (60 dB SPL^64,66,68^) and are, therefore, likely to be reflected in our experimental approach.

Taken together, the compensation mechanisms likely reflected in our results are critical for maintaining homeostasis, continued protection of peripheral structures, and preventing peripheral inhibition from degrading the encoding of important acoustic information. By contrasting short- vs. long-duration conditions, our results provide a potential non-invasive window into these mechanisms. As with any non-invasive markers, their true physiological origins must be established using direct, and likely invasive, studies of these systems. Specifically, future studies capable of selectively silencing the feedback from the T-stellate and small cells in the cochlear nucleus to the MOCR may provide the most conclusive evidence for our hypothesized gain compensation mechanism.

### Implications for research methods and the clinic

The MOCR function is important to consider in a clinical setting as it has been hypothesized to improve speech perception in noise^69–71^. Prior arguments for a MOCR role in speech perception were based only on its ability to restore the dynamic range of the auditory nerve^1,2,6,8^. Recent evidence suggests different feedback loops in the auditory pathway may respond differently to the type of auditory stimulus^55^. Evidence of MOCR collateral activity in the cochlear nucleus^21–24^—enhancing T-stellate and small cell output—further strengthens the role of MOCR in speech perception. OAEs are typically used to measure the MOCR strength in normal-hearing individuals. Given that OAEs rely on OHC activity, hearing loss due to OHC damage emphasizes the need for alternative measures of MOCR function. In addition to cochlear microphonics^33–35^, ASSRs appear to be a promising alternative for this purpose. Our findings indicate that the time scale and generation site at which inhibitory effects are compensated for must be carefully considered if ASSRs were to be used to deduce MOCR influence on the cochlear neural output. For instance, the contralateral noise-mediated inhibition of 20/40 Hz ASSRs^15–20^ may not reflect MOCR inhibition of OHC activity (Fig. 3A,B) as any reduction in peripheral input to the cortex is likely compensated for at the brainstem.

### If the brainstem compensates, why is inhibition still present in the brainstem and the cortex?

The inhibition of brainstem-generated (80 Hz) ASSRs observed in the short-duration condition reveals a coarse timeline to this compensation. Currently, there are no studies that directly describe the physiological time course of T-stellate/small cell-MOCR-mediated gain compensation to confirm our non-invasive findings. Nevertheless, the pattern of results in this study may be explained by the established kinetics of the MOCR pathway. It can be conjectured that inhibition of auditory nerve inputs by the MOCR causes an initial reduction in inputs to the cochlear nucleus, likely on a scale of several tens to a few hundred milliseconds, commensurate with the MOCR activation time course of around 0.25 s and the roughly 0.4 s it takes to reach steady state^31,32^. Considering that both the MOCR and T-stellate cells integrate energy over time, the gain compensation could happen over a few seconds. This initial reduction in peripheral input is reflected as reduced brainstem-dominated ASSR amplitude (80 Hz) in the short-duration condition. This is somewhat supported by the positive correlation with MOCR inhibition of OAEs and 80 Hz ASSR in this condition (Fig. 3D).

When contralateral noise was introduced, a reduction in cortex-generated (40 Hz) ASSR amplitude was observed with both long stimulus duration, consistent with previous studies^15–20^, as well as with short stimulus duration. However, the lack of significant correlation between the change in ASSR amplitude for 40 Hz and OAEs (Fig. 3A,B) suggests that the reduction in 40 Hz ASSR amplitude is unlikely to be related to the MOCR-mediated peripheral inhibition^15,20^. The reduction of 40 Hz ASSR amplitude may instead be explained by an interruption in thalamocortical loop resonance induced by contralateral noise. Desynchronization of 40 Hz ASSR, associated with a temporary reduction in the amplitude of oscillatory signal power in response to a concurrent stimulus, was similarly observed by Ross and colleagues^72,73^. ASSR desynchronization is a general reaction to both new and changing stimuli^72^ and is thought to act as a reset to the adaptation of auditory processing^74^. The reduction in 40 Hz ASSR amplitude may reflect this temporary desynchronization, which is more evident when averaging amplitude over short compared to long durations. This desynchronization, and the resulting robustness in the reduction of 40 Hz ASSR amplitude, could also explain the resistance to a potential adaptation-based compensation for cortically generated responses like that speculated for the 80 Hz/brainstem dominant ASSR (see also Discussion sub-section “Adaptation may restore brainstem-generated ASSR”).

In summary, our findings offer a potential new window into a gain compensation mechanism in the brainstem previously identified in animal models. Additionally, this study demonstrates the ability of the 80 Hz ASSR to measure MOCR function using short-duration stimuli. Our methods and corresponding results also emphasize the importance of timescale consideration for future research utilizing ASSRs to measure the MOCR effects on retrocochlear neural output.

## Methods

### Participants

Twenty young, clinically normal-hearing, adults participated in the study. Clinically normal hearing was established by an unremarkable otoscopic examination, bilateral hearing thresholds ≤ 20 dB HL at octave frequencies from 0.25 to 8 kHz (SmartAuD, Intelligent Hearing Systems [IHS], FL, USA), normal middle ear function as measured by tympanometry (Titan, Interacoustics, Denmark), measurable (magnitude greater than 0 dB with at least 6 dB signal-to-noise ratio [SNR]) distortion product OAEs (0.5–6 kHz at 65/55 dB SPL, SmartDPOAE, IHS, FL), and self-report of no neurological disorders. Two participants were rejected from analysis due to excessive noise in OAEs and two additional participants were rejected based on large middle ear muscle reflex (MEMR) activation (see MEMR sub-section below), reducing the number of participants to 16 (mean age ± standard deviation [SD]) = 23.4 ± 4 years; 1 male). Participants were either offered extra credit for their participation or compensated at the rate of $10/hour. The study procedures were approved by, and carried out following relevant guidelines and regulations of, the University of Wisconsin-Madison Health Sciences Institutional Review Board. Written informed consent was obtained from all participants prior to data collection.

### Stimuli

**Figure 6:**
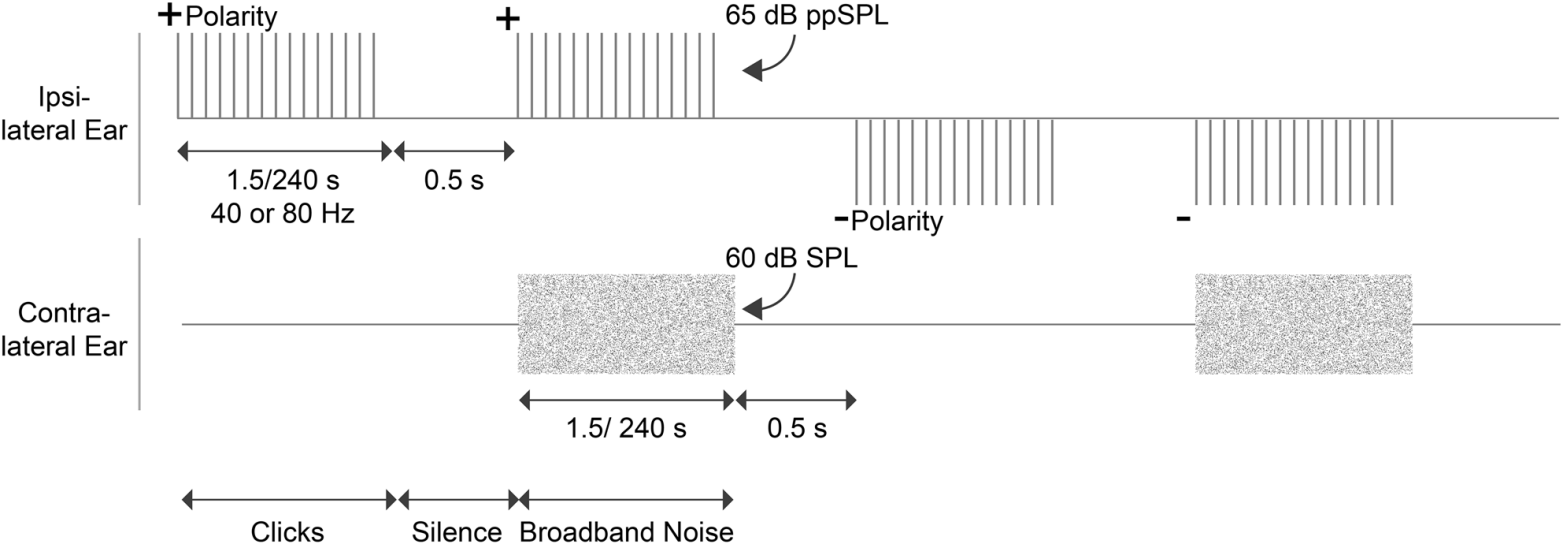
Schematic of the experimental protocol. Both 40 and 80 Hz clicks were presented in short (1.5 s) and long durations (4 min) at 65 dB ppSPL, with and without a 60 dB SPL broadband noise in the contralateral ear. Clicks were presented in positive and negative polarities to facilitate ASSR averaging. Each stimulus duration was separated by 0.5 s of silence.

All stimuli were digitally generated in MATLAB (v2017b; Mathworks, MA, USA) at a sampling rate of 96 kHz and a bit-depth of 24. The stimuli used to elicit OAEs and ASSRs were click trains with click rates of either 40 or 80 Hz presented at 65 dB peak-to-peak (pp) SPL. Whereas the 40 Hz clicks elicit a predominantly cortical response, the 80 Hz clicks elicit a predominantly brainstem response^75–78^. For brevity, although the 40 Hz and 80 Hz responses are referred to synonymously with cortical vs. brainstem sources, it is acknowledged that both scalp-recorded responses reflect multiple neural generators. The clicks were bandlimited between 0.8 and 5 kHz to focus the stimulus energy on frequency regions where the MOCR activity is most prominent when measured using OAEs^78,79^. Bandlimited clicks were generated in the frequency domain using a recursive exponential filter^80,81^ and inverse Fourier transformed to the time domain. The duration of the click was ∼108 μs. Clicks were presented in positive and negative polarities to reduce potential contamination with stimulus artifacts when averaging for ASSRs. Broadband noise (0.001 to 10 kHz) was presented at 60 dB SPL in the contralateral ear to elicit the MOCR. Both the ipsilateral clicks and the contralateral noise are illustrated in Fig. 6. In-ear calibration was performed for clicks to ensure the peak-to-peak (pp) level of the click stimulus was 65 dB ppSPL in all participants. Broadband noise was calibrated in an ear simulator (Type 4157, Bruel & Kjaer, Denmark).

### Instrumentation

Stimuli were generated, delivered, and controlled through an iMac computer (Apple, CA, USA) running Auditory Research Lab Audio Software (ARLas v4.2017^82^) on MATLAB. The iMac was interfaced with an external sound card (Fireface UFX+; RME, Germany) via Thunderbolt for analog-to-digital-to-analog conversion at a sampling rate of 96 kHz. Clicks were presented in the ipsilateral ear via one of the miniature loudspeakers of the ER10C (Etymotic Research, IL, USA) system. Ear canal pressures were registered and amplified (+ 20 dB) by the ER10C probe microphone placed in the ear. To avoid changes in stimulus level throughout the experiment, the probe placement was secured in the ear using foam tips and using silicone putty around the probe in-ear (Silicast, Westone Laboratories, CO^83^). The MOCR eliciting broadband noise was presented in the contralateral ear using an ER2 (Etymotic Research, IL) insert earphone coupled with a foam tip of appropriate size.

Electroencephalography (EEG) amplitude was registered by the Universal Smart Box (USB; IHS, FL, USA) controlled by a Windows desktop computer equipped with the Continuous Acquisition Module (IHS, FL, USA) at the sampling rate of 10,016 Hz. One of the IHS USB channels recorded triggers (5 V impulses) that coincided with the onset of stimulus blocks to index EEG data accurately. A single-channel montage was used for EEG acquisition with three sintered Ag–AgCl electrodes. The vertex (Cz) was used as the non-inverting electrode site and the nape was used as the inverting site^84^. The left collarbone was used as the ground. All electrode sites were cleaned with alcohol wipes and scrubbed with a mild abrasive gel (Nuprep; Weaver & Company, Aurora, CO) before affixing electrodes with adhesive sleeves and conduction gel (SignaGel; Parker Laboratories, Fairfield, NJ). Electrode impedances were monitored throughout the experiment and were always < 3 kΩ at each site.

### Experimental design

The experiment was conducted in a double-walled sound-attenuating booth where participants sat comfortably in a recliner for the duration of the experiment. Participants were instructed to sit relaxed, not move, swallow as few times as comfortable during stimulation, and maintain a wakeful state (watching a silent, closed caption movie). As shown in Fig. 6, OAEs and ASSRs were measured at 40 and 80 Hz with and without contralateral noise, in short (1.5 s) and long (240 s/4 min) durations. The conditions (rate and duration) alternated between clicks with and without contralateral noise separated by 0.5 s of silence and were repeated in positive and negative polarities. The long-duration condition was a single block of 240 s recording completed separately for with- and without-contralateral noise, i.e., no repetitions. In contrast, in the short-duration condition, 1.5 s-long click trains were repeated 160 times to match the total number of clicks presented in the respective long condition. This was done to avoid SNR differences in responses between long and short conditions analyzed in the frequency domain. The order of presentation in short/long conditions was counter-balanced but the 40 Hz short duration was always completed first to ensure maximum wakeful participant state as 40 Hz can be attenuated by sleep^84^. The ipsilateral/stimulus ear was chosen based on the ear with the largest DPOAE amplitude obtained during screening. The experimental procedure took about two hours with breaks, as desired by the participant, between conditions.

### Response analysis

OAEs were extracted from click ‘epochs’, defined as the duration between two successive clicks. OAE analysis was performed offline in MATLAB using custom scripts. First, stimuli with negative polarity were inverted. The clicks were sufficiently symmetrical such that the polarity inversion did not affect average OAE amplitudes. Next, the raw ear canal pressure recording was bandpass filtered around the click frequency (0.8–5 kHz). An artifact rejection routine was implemented where clicks with a root-mean-square (RMS) amplitude that fell outside the third quantile + 2.25 times the interquartile range (specific to the condition and within participants) were excluded from further analysis^31^. Typically, less than 10% of the responses were rejected across participants. The stimulus (0–4 ms) and OAEs (6.5–12.5 ms) were then separated for further analysis for both 40 and 80 Hz rates across stimulus durations and contralateral noise conditions. Specifically, OAEs were used to determine the presence of MOCR effects. The click stimulus was used to determine the presence of the middle ear muscle reflex (MEMR) effects that can potentially confound MOCR effects on OAEs^8,83^.

OAEs were first considered in the frequency domain to extract responses that were 12 dB above the noise floor. This step was done to ensure the MOCR-mediated inhibition was estimated only from high-quality OAEs^31,83,86,87^. Next, the responses were converted back to the time domain for averaging across the two buffers (even and odd-numbered epochs). OAE amplitude was estimated as the mean RMS of ear canal pressure between 6.5 and 12.5 ms across the two buffers and the noise floor was estimated as the mean difference between the two OAE RMS buffers. For each duration and rate condition, the RMS of OAE and stimulus magnitude (dB SPL) with contralateral noise were subtracted from the RMS without contralateral noise to compute the effect of MOCR and MEMR (in dB), respectively.

The same pre-processing strategy as ear canal pressure was applied to the raw EEG data. Raw EEGs were chunked into 1.5-s or 4-min epochs corresponding to short and long durations, respectively. Chunked EEGs were averaged over opposite stimulus polarities to minimize any stimulus artifacts. Finally, ASSR amplitudes were determined from the Fourier transforms of the averaged EEGs across with and without contralateral noise estimates at 40 and 80 Hz. EEG noise floor was estimated as the average of 8 frequency bins around the ASSR frequencies^84^. The reduction in response amplitude with contralateral noise relative to no-noise condition is referred to as ‘inhibition’ for both OAEs and ASSRs.

### Middle ear muscle reflex (MEMR) estimation

**Figure 7:**
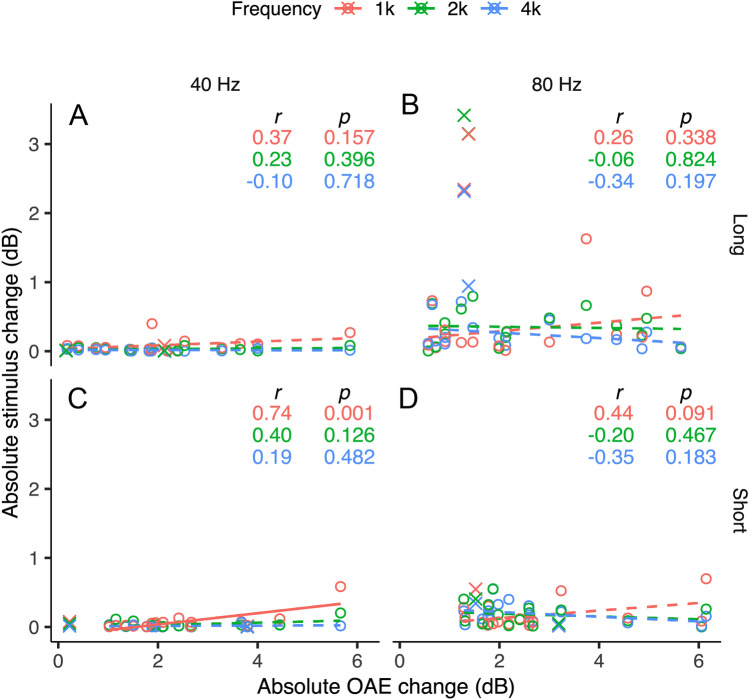
Stimulus vs. OAE change. Stimulus amplitude change as a function of OAE amplitude change for (**A**) 40 Hz click-rate, long stimulus duration (**B**) 40 Hz click-rate, short stimulus duration (**C**) 80 Hz click-rate, long stimulus duration (**D**) 80 Hz click-rate, short stimulus duration. Open circles represent individual participants. Colors indicate 1, 2, and 4 kHz 1/3^rd^ octave-band absolute stimulus change. A solid regression line represents a significant relationship between variables. A dashed regression line represents a non-significant relationship between variables. The resulting correlation coefficient (*r*) and *p*-value are presented in each panel.

When elicited by high-level sound, the MEMR stiffens the ossicular chain, altering signal transfer through the middle ear^83,88^ and may thus confound MOCR effects on OAEs^8,31,85,86^. The click stimuli (0–4 ms) in the same frequency range as the OAEs were analyzed to determine the presence of MEMR. The same analyses as OAEs were applied to obtain stimulus levels across conditions, except the responses were considered in three 1/3^rd^ octave bands centered around 1, 2, and 4 kHz. Multiple frequencies were considered separately because MEMR activation can increase or decrease stimulus reflectance in the ear canal in a frequency-specific manner^83,89^. To evaluate if the MEMR influenced OAE levels, we performed three tests. First, we identified outliers, i.e., individuals with large stimulus level changes. As evident in Fig. 7B, stimulus level changes are the largest and most variable for the 80 Hz long condition. Therefore, to avoid minimizing the size of the true outliers and rejecting data unnecessarily, we only used the 80 Hz long condition to identify outliers. Two participants, who were identified as outliers consistently in at least two of the three frequency bands, were rejected (crosses in all panels of Fig. 7). The remaining large stimulus changes in the 80 Hz long condition were not rejected because these changes occurred only in one frequency per participant and were not consistently observed across all three frequency bands. Secondly, a 3-way repeated measures analysis of variance (RM-ANOVA) was performed to test if the stimulus varied as a function of independent variables duration, click rate, and contralateral noise separately for the three frequency bands in the remaining 16 participants. Our results show that none of the main effects and interactions were significant (p > 0.05), except for the main effect of rate for the 4 kHz band (*F*[2, 15] = 8.2, *p* = 0.01), consistent with the apparently large stimulus level changes for 80 Hz rate in general in Fig. 7. The lack of a main effect of, and interactions with, the contralateral noise variable suggests that there was no systematic activation of the MEMR with contralateral noise.

However, stimulus changes due to contralateral noise do not necessitate a systematic influence of MEMR on OAE measurements^31^ and, as such, MOCR estimation. Therefore, to evaluate the relationship between stimulus level changes and OAE level changes, thirdly, we performed Pearson correlations. Considering the stimulus changes can be both positive and negative, especially across frequencies, we performed correlations using absolute stimulus and OAE changes. As seen in Fig. 7C, only the positive correlation for the 40 Hz short condition for the 1 kHz band is significant, possibly driven by 1 data point. This same trend is not replicated in other frequency bands or in the 1 kHz 80 Hz condition, where a larger MEMR activation would be expected resulting from greater stimulus energy integration per unit time. Taken together, ANOVA and correlations across frequencies are not consistent with the MEMR systematically influencing the results presented herein. Therefore, the OAE changes and group effects presented in this study are predominantly driven by the MOCR. Despite the lack of evidence for a systematic MEMR influence on MOCR measures, as with any human MOCR study, small and non-significant MEMR effects potentially influencing MOCR effects cannot be categorically ruled out.

## Data Availability

All data generated and analyzed during this study are included in this published article across figures.
